# Selenium, Copper, Zinc Concentrations and Cu/Zn, Cu/Se Molar Ratios in the Serum of Patients with Acute Ischemic Stroke in Northeastern Poland—A New Insight into Stroke Pathophysiology

**DOI:** 10.3390/nu13072139

**Published:** 2021-06-22

**Authors:** Anna Mirończuk, Katarzyna Kapica-Topczewska, Katarzyna Socha, Jolanta Soroczyńska, Jacek Jamiołkowski, Alina Kułakowska, Jan Kochanowicz

**Affiliations:** 1 Department of Neurology, Medical University of Bialystok, M. Skłodowskiej-Curie 24a, 15-276 Białystok, Poland; katarzyna-kapica@wp.pl (K.K.-T.); alakul@umb.edu.pl (A.K.); kochanowicz@vp.pl (J.K.); 2 Department of Bromatology, Faculty of Pharmacy with the Division of Laboratory Medicine, Medical University of Białystok, Mickiewicza 2D, 15-222 Białystok, Poland; katarzyna.socha@umb.edu.pl (K.S.); jolanta.soroczynska@umb.edu.pl (J.S.); 3 Department of Population Medicine and Lifestyle Diseases Prevention, Medical University of Bialystok, M. Skłodowskiej-Curie 24a, 15-276 Białystok, Poland; jacek.jamiolkowski@umb.edu.pl

**Keywords:** trace elements, ischemic stroke, selenium, zinc, copper, Cu/Zn molar ratio, Cu/Se molar ratio, antioxidants

## Abstract

Background: In recent years, trace elements (TEs) have gained considerable attention in the course of treatment and diagnosis of ischemic stroke. The purpose of the conducted research was to determine the trace mineral status (Se, Cu, Zn, Cu/Zn ratio, and Cu/Se ratio) in patients with acute ischemic stroke compared to the population of healthy people in the northeastern region of Poland. Materials and methods: 141 patients with acute ischemic stroke (AIS) and 69 healthy control subjects were examined. The serum concentrations of mineral components were assessed by the atomic absorption spectrometry method. Clinical parameters were updated based on medical records. Results: The serum Se and Zn concentrations were significantly decreased (*p* < 0.0001; *p* < 0.0001) in patients with AIS compared with healthy control subjects. However, no significant differences were revealed in terms of the serum Cu concentration (*p* = 0.283). As expected, we found that the serum Cu/Zn and Cu/Se molar ratios were significantly higher (*p* = 0.001; *p* < 0.0001) in patients with AIS compared with healthy control subjects. Conclusions: Disturbed metal homeostasis is a significant contributor to AIS pathogenesis. Furthermore, marked disruption of the serum Cu/Zn and Cu/Se molar ratios could serve as a valuable indicator of AIS patients’ nutritional status and oxidative stress levels.

## 1. Introduction

Stroke is the second leading cause of death worldwide, following only ischemic heart disease, and is one of the main causes of long-term disability across the globe, with its impact ever increasing [1,2,3]. According to a European report in 2017 [4], the incidence rate for all strokes in Poland was 112/100,000 population, with mortality rates of 69.3/100,000. In recent years, trace elements (TEs) have gained considerable attention in the treatment and diagnosis of ischemic stroke. TEs are essential for maintaining human health due to their involvement in numerous signaling pathways and metabolic processes [5]. Some studies have shown that TE deficiencies in ischemic stroke occur more commonly than previously suspected [6,7,8,9,10,11,12,13,14,15,16,17].

Zinc (Zn) and copper (Cu) are the most common metals in the human body, with relatively large amounts found in the brain. While their functions in the inflammation process are yet to be fully explored, it has been shown that they are essential in controlling the synthesis of free oxygen radicals because of their involvement in antioxidant stress modulation. Zn has many known functions and its prevention of free radicals has been the focus of numerous studies [18,19,20]. It is thought that an elevated fraction of free cooper in serum may be harmful due to its significant oxidation–reduction potential through the generation of reactive oxygen species in Fenton and Haber–Weiss type reactions [21]. They are essential to the operating of the immune system and proper functioning of a variety of physiological and biochemical processes [22,23]. Apart from being a cofactor for numerous enzymes, they play the key role in the development and functioning of the central nervous system [24,25,26]. Disturbed homeostasis and distribution of TEs related to anti-oxidant, anti-inflammatory, and apoptotic effects seem to be connected with neurodegenerative diseases and aging [27]. Disturbances in Cu homeostasis lead to impaired neuronal function and neurological diseases, including Alzheimer’s disease and Wilson’s disease [21]. Recent observation has suggested that Zn deficiency might contribute to the accumulation of senescent cells and to vascular pathology as well as ischemic stroke [28].

Selenium (Se) is an essential mineral that is critically involved in immunogenic, anticarcinogenic, and antimutagenic processes, including cell proliferation control and anti-aging activities. Se and many selenoproteins, as components of the antioxidant system, may exhibit neuroprotective properties [14,29,30]. The up-to-date human data have suggested that stroke is connected with considerably decreased Se levels, thus some studies have indicated the importance of preventing Se deficiencies in patients with risk factors for cardiovascular diseases, including ischemic stroke [14,31,32].

Antioxidants, including enzymatic cofactors such as Zn, Cu, and Se, may serve as indicators showing oxidative stress defense [33,34,35]. Collecting data on Se, Zn, and Cu in stroke pathophysiology is still a challenge. Analysis of various biomarkers with prognostic value can provide a potential treatment. The objective of this study was to assess the trace mineral status (Se, Cu, Zn, Cu/Zn ratio, and Cu/Se ratio) in patients with acute ischemic stroke compared to a population of healthy people in the northeastern region of Poland and the correlations between TEs and the patients’ clinical data. As far as we are aware, this is the first study investigating the trace mineral status in patients with AIS in a Polish population.

## 2. Materials and Methods

This clinical study was conducted between January 2019 and November 2020 in the Department of Neurology, Medical University of Bialystok (UMB). We enrolled 141 consecutive AIS patients (60 patients with AIS treated with intravenous thrombolysis and/or mechanical thrombectomy and 81 patients who had undergone conservative treatment).

Inclusion criteria included: age 18–85 years, hospitalization up to 24 h following the presence of neurological symptoms; neuroimaging: computed tomography (CT) and magnetic resonance imaging (MRI) to determine the size of the infarction and exclude intracranial and subarachnoid hemorrhage stroke and tumors. All participants were recruited 3–5 days following symptom onset.

The exclusion criteria were: recent history (<4 weeks) of myocardial infarction and acute surgery, trauma; previous stroke; acute inflammatory and infectious diseases during last month; autoimmune diseases (rheumatic disease) [36,37], advanced heart failure, chronic kidney disease stage 5, and/or liver insufficiency; active malignant cancer; recent (up to 3 months before hospitalization) intake of mineral supplements.

The 69 healthy control subjects without stroke and chronic cerebrovascular diseases were recruited among volunteers from the general population who applied to the Department of Bromatology, UMB.

The demographic, clinical, cardiovascular risk factors (arterial hypertension, smoking status, diabetes, alcohol abuse, dyslipidemia, previous heart diseases, atrium fibrillation, and history of prior stroke), medication history, and laboratory data were analyzed. The neurological condition was assessed using the National Institutes of Health Stroke Scale (NIHSS) at admission and discharge [38] and the modified Rankin Scale (mRS) [39] at discharge. The etiology of ischemic stroke was defined in line with the TOAST (Trial of Org 10,172 in Acute Stroke Treatment) [40] classification based on neurologic examination and CT or MRI of the brain, the results of external B-mode ultrasound carotid imaging, head and/or neck CT angiography, 12-channel ECG, and echocardiography. Body mass index (BMI) was defined as weight in kilograms (kg) divided by height in meters squared (m^2^).

The protocol of the study was accepted by the Ethics Committee of the Medical University of Bialystok (R-I-002/276/2018). Written informed consent was collected from all study participants or next of kin prior to the collection of blood samples. Clinical parameters were updated based on medical records.

### 2.1. Blood Sample Collection and Analysis

Blood samples (5 mL) were obtained from all study participants with the use of vacutainer system test tubes with a clot activator. The samples were drawn once: within 3 to 5 days after the onset of neurological symptoms. The samples were then centrifuged at 2500 rpm for 15 min at room temperature to separate the serum. The serum samples were aliquoted into microtubes and stored at −80 °C prior to being inspected for determination of Cu, Zn, and Se at the Department of Bromatology, UMB. All reagents and chemicals used in the study were presented at an analytical grade for spectral analysis.

To determine the concentration of Cu and Zn, serum samples were deproteinized with 1 mol/L Nitric acid prepared from 69% Suprapur^®^, Merck, Darmstadt, Germany. Next, 1% Triton™ X-100, Sigma-Aldrich, St. Louis, MO, USA, was included as a surfactant, and the samples were centrifuged at 2000 rpm for 10 min. The concentration of Zn was calculated in the supernatant. In the case of Cu, the samples were diluted with 0.1 mol/L nitric acid. To determine Se concentration, serum samples were directly diluted with 0.2% Triton X-100. Standard solutions of Cu, Zn, and Se for calibration curves were prepared from concentration 1000 mg/L, Merck, Darmstadt, Germany. In order to eliminate any dust particles, all plastic materials (tubes, pipette tips) were washed in 5% nitric acid for 24 h, then they were washed 3 times with distilled water and 6 times with ultrapure water and dried at 50 °C.

The concentration of Zn was determined by atomic absorption spectrometry with air-acetylene flame atomization, with Zeeman background correction. Wavelength: 213.9 nm, slit width (nm): 1.3, lamp current (mA): 5.0. Concentrations of the standard solutions for the calibration curve: 0.5, 1.0, and 1.5 mg/L. The concentration of Cu was determined by atomic absorption spectrometry with electrothermal atomization, with Zeeman background correction. Wavelength: 324.8 nm, slit width (nm): 1.3, lamp current (mA): 7.5. Concentrations of the standard solutions for the calibration curve: 10, 20, and 40 µg/L. Analytical conditions: dry (start temp./end temp.): 80/140 °C, ramp time: 40 s; ash: 800/800 °C, ramp time: 20 s; atomize: 2400/2400 °C, hold time: 5 s; clean: 2500/2500, hold time: 4 s. The concentration of Se was determined by atomic absorption spectrometry with electrothermal atomization, with Zeeman background correction and using a palladium–magnesium matrix modifier—Pd: 1500 ppm and Mg: 900 ppm (palladium matrix modifier, Merck, Darmstadt, Germany, magnesium nitrate, Sigma-Aldrich, St. Louis, MO, USA). Wavelength: 196 nm, slit width (nm): 1.3, lamp current (mA): 12.5. Analytical conditions: dry (start temp./end temp.): 80/140 °C, ramp time: 40 s; ash: 900/900 °C, ramp time: 30 s; atomize: 2500/2500 °C, hold time: 5 s; clean: 2700/2700, hold time: 4 s. Concentrations of the standard solutions for the calibration curve: 10, 25, and 50 µg/L. In each case, mono-element lamps were used.

Certified reference material of human serum (Seronorm Trace Elements, Serum L-1, SeroA, Billingstad, Norway) was used to investigate the reliability of this approach. The findings of the quality control analyses were equivalent to the reference values.

The accuracy of the method was 1.7%, 1.3%, and 1.4% and the coefficient of variation was 3.9%, 2.5%, 2.7% for Se, Cu, and Zn, respectively. The detection limit of the methods was 1.71 µg/L, 0.00058 mg/L, and 0.011 mg/L for Se, Cu, and Zn, respectively. The biochemical assays were conducted in accordance with the standard protocols, and the values of Zn and Cu are presented in mg/L and for Se in μg/L.

The Department of Bromatology of UMB participates in the trace elements analysis quality control program supervised by the National Institute of Public Health, the National Institute of Hygiene, and the Institute of Chemistry and Nuclear Physics. Cu, Zn, and Se concentrations, after calculation in mmol/L, were used to indicate the metal dyshomeostasis by evaluating the Cu to Zn ratio and the Cu to Se ratio. The concentration of mineral components in the serum and molar ratio between Cu and Zn and Cu and Se were estimated and compared among patients with AIS and the control group. Plasma selenium and copper were acknowledged as reliable biomarkers for chronic selenium or copper exposure [6]. Additionally, selected plasma levels of basic biochemical parameters were determined in the accredited Biochemical Clinical Laboratory of the University Clinical Hospital in Bialystok. The levels of the measured parameters were contrasted with the reference values of this laboratory.

Each patient had a fasting lipid profile, comprising total cholesterol (TC), triglycerides (TG), high-density lipoprotein cholesterol (HDL-C), and low-density lipoprotein (LDL-C) values. Serum lipid concentrations were measured by enzymatic methods. Concentrations are represented in mg/dL.

### 2.2. Statistical Analysis

Statistical analyses were performed using IBM SPSS Statistics 27.0 [41] and R software 4.0.3 [42]. The normality of the distribution of quantitative variables was assessed using the Shapiro–Wilk test. Due to statistically significant deviations from the normal distribution of most variables, nonparametric methods were used in the analysis. The two groups were compared using the Mann–Whitney test. In the case of comparing more subgroups, the Kruskal–Wallis test was applied, and when significant differences were found, tests for all pairs according to Dwass–Steele–Critchlow–Fligner were performed [43]. Correlations between the pairs of quantitative variables were assessed using the Spearman’s rank-order correlation. Dependencies between qualitative variables were tested using Pearson’s χ^2^ independence tests. Statistical hypotheses were verified at α = 0.05 significance level.

## 3. Results

We studied 141 consecutive patients with AIS (including 60 patients undergoing interventional management and 81 patients with conservative treatment) and 69 healthy control subjects.

Arterial hypertension was found in over 90% of patients with AIS. More than 81% of patients with AIS had abnormal findings on extracranial carotid sonography (carotid intima-media thickness (CIMT) protrusion of >1.5 mm into the lumen or a focal intimal medial thickening of larger than 50% of the area surrounding the vessel). According to the clinical data, brain lesions more commonly occurred in the left hemisphere (54% of AIS patients) than in the right hemisphere. No statistical differences between patients and control subjects concerning male and female distributions were observed (*p* = 0.251). The baseline general demographic characteristics, biochemical values, and the serum levels of Se, Cu, Zn, and Cu/Zn and Cu/Se molar ratios in the patients with AIS and control subjects are presented in Table 1 and Table 2.

**Figure 1:**
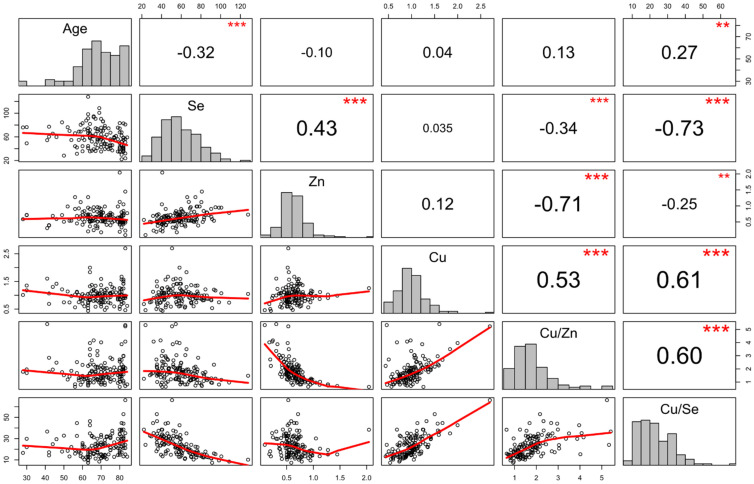
Spearman’s rank-order correlation matrix with scatterplots for serum TEs and molar ratio levels of these elements in the patients with acute ischemic stroke. *p*-values < 0.05 were considered statistically significant; ** *p* < 0.01, *** *p* < 0.001.

We found positive correlations between the concentrations of Cu and the Cu/Zn, Cu/Se ratios (r = 0.53, *p* < 0.001; r = 0.61, *p* < 0.001), as well as the Se and Zn concentrations (r = 0.43, *p* < 0.001) and Cu/Zn and Cu/Se molar ratios (r = 0.60; *p* < 0.001) in patients with AIS. Age was positively related to the Cu/Se molar levels and negatively to Se concentrations in patients with AIS (r = 0.27; *p* = 0.001, r = −0.32; *p* < 0.001, respectively). We observed negative correlations in patients with AIS between concentrations of Zn, Se, and the Cu/Zn molar ratio (r = −0.71, *p* < 0.001; r = −0.34, *p* = 0.001, respectively), as well as Zn, Se, and the Cu/Se molar ratio (r = −0.25, *p* = 0.003; r = −0.73, *p* < 0.001, respectively) (Figure 1). The significant negative correlations were observed between the BMI index and Cu, Cu/Zn ratio, and Cu/Se ratio in the patients with AIS (*p* = 0.048, *p* = 0.048, *p* = 0.018, respectively).

**Figure 2:**
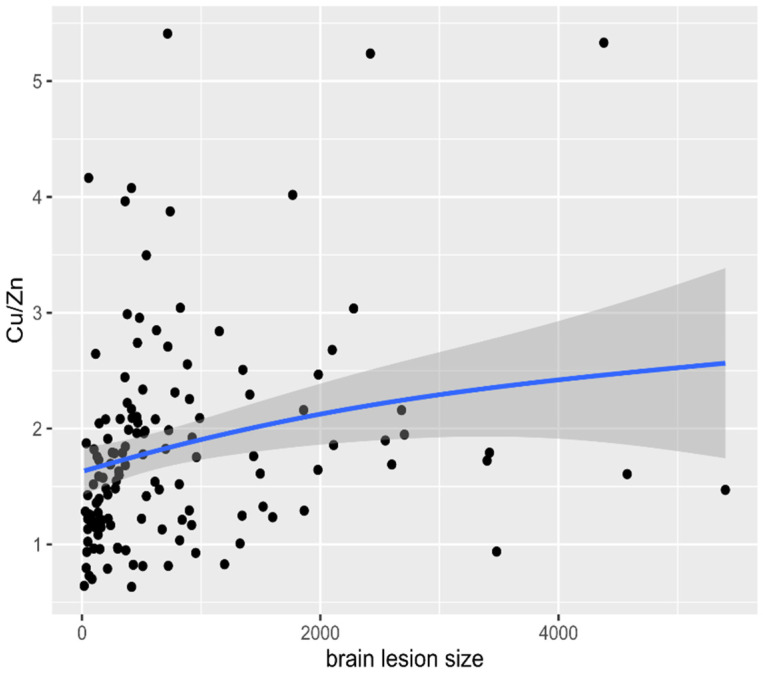
Correlation between brain lesion size in neuroimaging (head CT/MRI) with Cu/Zn molar ratio levels in patients with acute ischemic stroke (AIS). Abbreviations: Cu, Copper. Zn, Zinc. CT, Computered Tomography. MRI, Magnetic Resonance Imaging.

**Figure 3:**
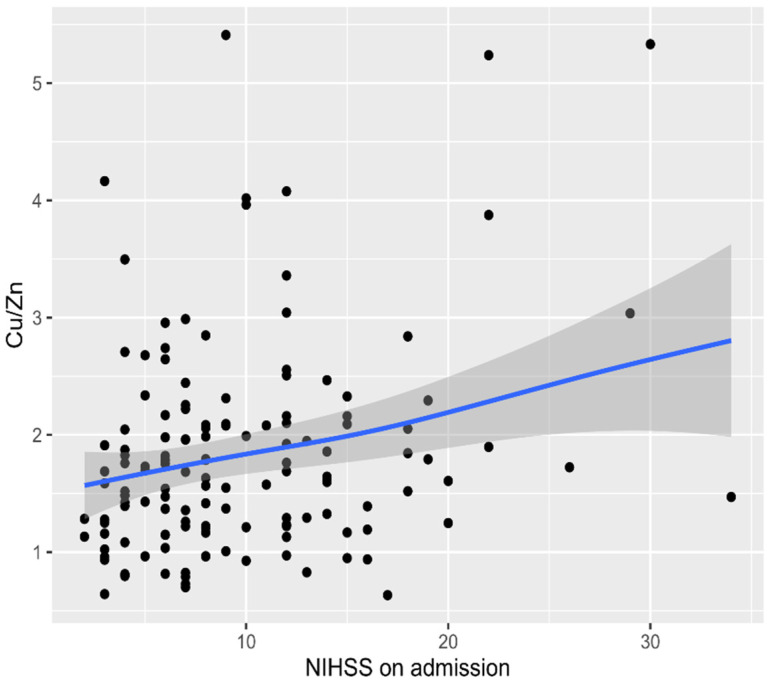
Correlation between the Cu/Zn molar ratios and the National Institutes of Health Stroke Scale (NIHSS) values on admission in patients with acute ischemic stroke (AIS). Abbreviations: Cu, Copper. Zn, Zinc.

The following correlations were observed: a positive correlation between brain lesion size in neuroimaging (CT/MR) with Cu, Cu/Zn molar ratio (Figure 2), but negative correlation with Zn concentration in patients with AIS (r = 0.19, *p* = 0.0033; r = 0.35 *p* < 0.001; r = −0.22, *p* = 0.011 respectively). The obtained data indicated statistically significant correlations between the Cu/Zn molar ratio and the NIH value on admission (r = 0.21, *p* = 0.014) (Figure 3), and between Zn and NIHSS level on admission (r = −0.21, *p* = 0.015).

**Figure 4:**
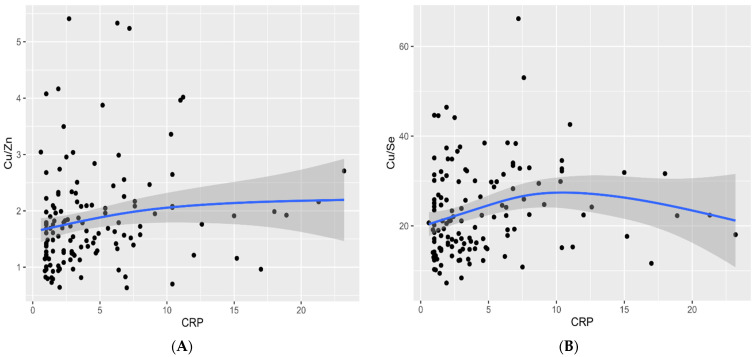
Correlation between Cu/Zn (**A**) and Cu/Se (**B**) molar ratio levels and CRP serum values (mg/L) in patients with acute ischemic stroke (AIS). Abbreviations: Cu, Copper. Zn, Zinc. Se, Selenium. CRP, C-reactive protein.

Furthermore, we noticed a strong correlation between brain lesion size and NIHSS on admission (r = 0.70, *p* < 0.001). The Cu/Zn ratio increased as the severity of neurological manifestations (NIHSS) progressed with no significant correlation in patients’ functional status at discharge evaluated by the MRS (*p* = 0.208). We observed that higher Cu serum levels and Cu/Zn (Figure 4A) and Cu/Se molar ratios (Figure 4B) were associated with elevated CRP values (r = 0.28, *p* = 0.006; r = 0.24, *p* = 0.018; r = 0.24, *p* = 0.004, respectively). The results confirmed two associations concerning TG and Se concentrations (r = 0.18, *p* = 0.032) and TC and Se concentrations (r = 0.22, *p* = 0.008).

**Figure 5:**
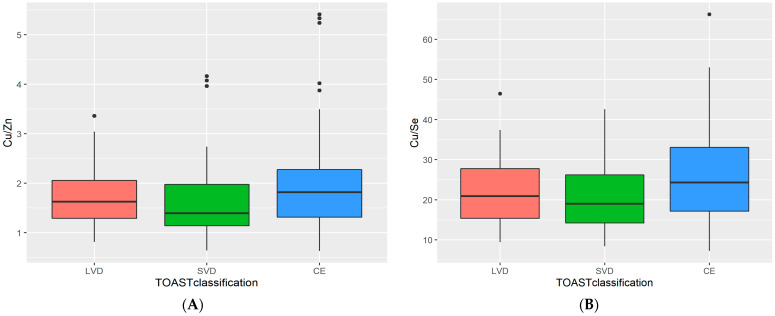
Box plot depicting the results for serum Cu/Zn (**A**) and Cu/Se (**B**) molar ratio levels in patients with acute ischemic stroke in relation to the The TOAST (trial of ORG 10172 in acute stroke treatment) classification. The cardioembolic group showed higher values both in Cu/Zn and Cu/Se molar ratios in patients with AIS. Abbreviations: Cu, Copper. Zn, Zinc. LVD, Large-vessel disease. SVD, Small-vessel disease. CE, Cardioembolic.

We also evaluated the associations of plasma metal concentrations with traditional stroke risk factors. Statistically higher values of Cu/Zn molar ratios were observed in patients with AIS and atrial fibrillation (Me: 1.86, IQR: 1.11 vs. Me: 1.55, IQR: 0.76, respectively) (*p* = 0.006). The prevalence of diabetes mellitus type 2 was associated with lower values of Cu/Zn molar ratios (Me: 1.42, IQR: 0.84 vs. Me: 1.74, IQR: 0.96, respectively) (*p* = 0.037) and higher Zn concentrations (*p* = 0.006). A difference was identified in serum concentrations of Cu/Se ratio and Se with reference to the occurrence of hyperlipidemia (*p* = 0.017; *p* = 0.022). The statistically significant differences were observed in Cu/Zn and Cu/Se molar ratios in relation to the TOAST classification, and between the SVD and CE etiologies of ischemic stroke (*p* = 0.033; *p* = 0.026, respectively) in patients with AIS. The CE group showed higher values both in Cu/Zn and Cu/Se molar ratios (Figure 5A,B). There was a positive correlation between the severity of carotid arteriosclerosis and Zn concentration in non-smoking patients (r = 0.22, *p* = 0.048).

No statistically significant differences were observed in Se, Zn, Cu, and Cu/Zn and Cu/Se molar ratios in relation to the brain lesion location or the coexistence of hypertension in patients with AIS (*p* > 0.05). The administered treatment (intervention therapy compared to conservative) in patients with AIS had no impact on the concentration of Se, Zn, Cu, and Cu/Zn and Cu/Se molar ratios (*p* = 0.190; *p* = 0.919; *p* = 0.198; *p* = 0.605, *p* = 0.861 respectively).

The serum Se and Zn concentrations (μg/L, mg/L) were significantly decreased (*p* < 0.0001; *p* < 0.0001) in patients with AIS compared with healthy control subjects. However, no significant differences were found between patients with AIS and the healthy control subjects in relation to the serum Cu concentration (mg/L) (*p* = 0.283). As expected, we found that the serum Cu/Zn and Cu/Se molar ratios were significantly higher (*p* = 0.001; *p* < 0.0001) in patients with AIS compared with healthy control subjects (Table 2).

Significant differences in Cu levels were found between men and women in the healthy control subjects (*p* = 0.008). Additionally, women in the healthy control subjects were characterized by significantly higher Cu/Zn and Cu/Se molar ratios levels compared to men (*p* = 0.022; *p* = 0.037, respectively) (Table 2). Interestingly, the analysis showed positive correlations between the concentrations of Cu and the Cu/Zn ratio, Cu/Se ratio, and Se concentrations (r = 0.79, *p* = <0.001; r = 0.75, *p* = <0.001; r = 0.24; *p* = 0.049), as well as between the Cu/Se and Cu/Zn molar ratios (r = 0.67; *p* < 0.001) in healthy control subjects. We observed negative correlations in healthy control subjects between concentrations of Zn and Cu/Zn ratio (r = −0.45, *p* < 0.001) and Se and Cu/Se ratio (r = −0.40, *p* = 0.001). We observed lower Cu concentrations in older patients in the healthy control subjects (r = −0.31; *p* = 0.01).

## 4. Discussion

The most significant observation regarding the homeostasis of TEs in our study was the marked decrease in serum Se and Zn levels with the high concentrations of Cu/Zn and Cu/Se molar ratios in patients with AIS. This may be the likely effect of the acute inflammatory processes and oxidative stress resulting from ischemic stroke. Currently, in Poland, there have been no studies on the concentration of Se, Cu, Zn, and the Cu/Zn and Cu/Se molar ratios in the serum of patients with AIS. These results are in line with studies from other countries [7,8,9,11,16,45,46].

Se’s protective properties in ischemic stroke were primarily based on its antioxidative and detoxification effects. Previous cohort studies have demonstrated a solid bond between lower blood Se levels and the occurrence of hypertension, coronary heart disease, and ischemic stroke [11,46,47,48,49,50]. In addition, some previous studies have shown a positive trend between higher blood Se levels and the occurrence of diabetes, metabolic syndrome, and dyslipidemia [13,50,51,52,53,54,55,56,57]. Selenium deficiency has been reported in Polish patients with multiple sclerosis and pancreatic cancer [58,59,60], whereas no studies have been published with regard to patients with AIS in the adult Polish population. A cross-section study conducted on the Canadian population found an association between high blood/dietary Se levels and lower stroke prevalence [47]. Some studies indicated the importance of preventing Se deficiencies in patients with risk factors for cardiovascular diseases [31,32].

The negative relationship between Se concentration and CVD was reported in Chinese and European populations with low selenium exposure [7,11,61,62]. On the other hand, these results were not confirmed in other studies conducted on populations with a higher Se intake [15,63]. Some studies have shown that ischemic stroke was connected with a significant increase in Se levels in the serum [13,15,64]. Increased Se level may be a compensatory reaction aimed at reducing brain damage induced by ischemia. There are increasing data on Se’s potential neurotoxic effect at high exposure levels [65]. The reasons for increased Se levels in stroke-related brain damage are still unknown [17,66]. Previous studies have shown a U-shape dose response, which means that adverse effects are caused by both very low and very high Se levels [17].

Cu is an essential, albeit toxic, TE, which has a confirmed association with the risk of ischemic stroke [24]. There have been studies [6,8,9,13,46,48,67,68] that suggested that patients with AIS had elevated serum Cu concentration. A recent meta-analysis suggested that exposure to single metals (arsenic, lead, and Cu) could be connected to increased risk of CVD [69]. One study has shown an association between increased dietary Cu intake and a greater risk of stroke mortality [70]. Lower serum Cu levels can prevent brain damage resulting from oxidative stress following ischemic stroke [40]. In one study, patients with less successful clinical recovery showed increased Cu levels [71]. This may suggest the involvement of Cu with the plasticity related to stroke recovery. In our study, there was no correlation between Cu and NIHSS and/or MRS scale in patients with AIS. The inconsistent results in serum Cu analysis indicate that there is a need for further research.

In our study, we confirmed reduced serum Zn level in patients with AIS, which was in line with other studies [7,9,45,72]. Qi Z et al. reported that Zn is involved in blood–brain barrier damage after cerebral ischemia. Additionally, excessive Zn release and accumulation in microvessels has contributed to ischemia-induced neuronal and vascular injury [73]. Tomas-Sanchez et al. [74] discovered that lower doses of Zn may have a neuroprotective effect against cerebral ischemia. On the other hand, the accumulation of Zn leads to cytotoxicity, neuroinflammation, and neuronal death.

Xiao Y et al. [6] observed that higher plasma Cu levels were connected with increased stroke risk due to large artery atherosclerosis. In our study, we found that patients with cardioembolic stroke showed higher values in both Cu/Se and Cu/Zn molar ratios (Figure 5A,B). BMI is related to the impact of obesity on TE homeostasis [75]. A close association has been shown between Zn and cortisol, diabetes mellitus type 2, and obesity in recent studies [76,77]. However, our study revealed that patients with AIS and lower BMI index have higher serum Cu concentrations and Cu/Se and Cu/Zn molar ratios. It appears that metabolic stress in the course of obesity and metabolic syndrome causes a compensatory reaction characterized by an increased Zn-induced antioxidant protection mechanism [78,79]. These findings were noticed in our study.

The current literature seems to indicate a link between the metabolism of Cu and Zn. It has been established that TEs interact with each other, as seen in the strong competition between Cu and Zn. They are all bivalent ions, and as such, although of different sizes, they probably compete in transport through channels and carriers [80]. Some studies have suggested that elevated blood Cu and decreased blood Zn concentration were unrelated risk factors for cardiovascular disease [81,82]. The same was observed in our analysis. Noshin et al. [83] reported a decreased Zn/Cu ratio in CAD patients. Wen et al. [7] found that patients with AIS demonstrated elevated plasma Cu levels and decreased plasma Zn concentrations compared to the controls, but without statistically significant differences between the two groups. We decided to find the Cu/Zn ratio in the assessment of the relationship between Cu and Zn rather than the concentration of either of the two TEs. We confirmed a strong positive correlation between the Cu/Zn ratio and Cu level as well as a negative correlation between the Cu/Zn ratio and Zn level (Figure 1). There is remarkably scarce evidence in the literature to evaluate the performance of the antioxidant defense system, which should be conversely related to the size of the infarct and neurological condition [84]. In our study, we confirmed that during the acute phase of ischemic stroke, the immune response was associated with poor outcome, and an elevated Cu/Zn ratio was independently associated with a higher NIHSS scale at admission (Figure 3). Higher Cu/Zn molar ratio was observed in patients with larger brain infarct size (Figure 2). This study showed that the Cu/Zn ratio is likely to become a valuable marker for immune dysfunction in AIS and may have the potential to become a useful marker of oxidative stress and inflammation in the pathogenesis of AIS. Therefore, it is of key importance to maintain the homeostatic balance of TEs. More importantly, the need to establish TE thresholds and promote supplementation campaigns exists [85,86].

It was presupposed that there is a link between Cu and Se, which may be mediated by the protein product of SELENBP1 [60]. In a cohort study conducted by Cabral et al., the Se to Cu ratio was found to be the most sensitive CVD risk parameter [75]. We observed that Se concentrations and Cu/Se ratios may precisely show both oxidative stresses and increased inflammatory response in patients with AIS. Moreover, our findings are in line with other studies, which have shown a significant positive correlation between CRP and Cu and Cu/Zn ratio levels [13,87].

It has been suggested in earlier studies that Zn deficiency contributes to the development of CVDs [77,88], because of involvement in the pathogenesis of atherosclerosis [89]. It has been suggested that there is a relationship between plasma Cu concentrations and increased prevalence of hyperlipidemia [6,13,48]. While the precise relationship between Se and dyslipidemia remains to be fully known, Se likely plays a role in dyslipidemia with an impact on insulin sensitivity, inflammation reaction, and oxidative stress [13,57,90,91]. Our study found that patients with AIS and dyslipidemia have elevated Se concentrations, and therefore reduced Cu/Se ratio values. Furthermore, TC and TG increase significantly with elevated Se levels in serum, while increased Cu/Se ratio was inversely correlated with TC concentrations in patients with AIS. The observed alterations in Se, Zn, and Cu concentrations need further research to establish their use as independent biomarkers of atherosclerosis in patients with AIS.

TE homeostasis is affected by multiple factors such as age, sex, diet, and health status. Se and Zn concentrations are decreased in older adults [92]. There are reports of elevated levels of Cu combined with low levels of Zn in plasma in older adults. Some researchers believe that the Cu/Zn ratio is a biomarker of aging [5,18,92]. The presented results of the study were confirmed in our own study, which revealed the following: decreased Se concentrations, thus higher Cu/Se molar ratio levels in older patients with AIS. Furthermore, significant differences in Cu levels were found between men and women in the healthy control subjects. Additionally, women in the healthy control subjects were characterized by significantly higher Cu concentrations and Cu/Zn and Cu/Se molar ratio levels compared to men (Table 2). Interestingly, we observed lower Cu concentrations in older patients in the healthy control subjects.

Our study has several limitations. The studied sample was relatively small and all of the tests performed at a single institution. The study presented here was conducted in a single department and we could only obtain data from a one-point measurement from each patient. The results we obtained do not necessarily reflect the epidemiological condition of the population of Poland. Various drugs, including antiplatelet, angiotensin II type 1 receptor blockers, statins, and antidiabetic medication may affect TE concentration in patients with AIS. The study was limited to assessing differences over longer periods and could not monitor parameters from before the ischemic stroke event. Therefore, it was unable to show the specific change of serum TE levels. Finally, it is necessary to conduct more cohort studies in the future in order to confirm the associations identified over the course of this study. Despite these limitations, this study provides valuable insights in the search for biomarkers that could be useful in screening high risk factors of ischemic stroke. Also, to the best of our knowledge, this is the first study that established the relationship between serum TEs and ischemic stroke in the Polish population.

## 5. Conclusions

Our study showed that disturbed metal homeostasis is a significant contributor to AIS pathogenesis. Furthermore, marked disruption of the serum Cu/Zn and Cu/Se molar ratios could serve as a valuable indicator of AIS patients’ nutritional status and oxidative stress levels. Changes in TE levels in the serum can be used as a prognostic biomarker in patients with AIS. There is a need for additional research to explore the possible effects of TE supplementation in ischemic stroke management, as well as to establish dietary TE reference values for stroke prevention. 

## Figures and Tables

**Table 1:** Demographic data and biochemical values of study population.

Clinical Parameters	AIS (*n* = 141)	Controls (*n* = 69)	p *
Gender [M *n*(%) / F *n*(%)]	67 (47.5%) / 74 (52.5%)	27 (39.1%) / 42 (60.9%)	0.251
Age (years) median (Q1-Q3)	70 (63–79.5)	55 (38.5–65.5)	<0.05
BMI (Kg/M^2^) median (Q1-Q3)	26.83 (24.23–30.08)	27.19 (23.26–29.10)	0.519
Total cholesterol (TC) (mg/dL) median (Q1-Q3)	179 (143–212)		
Triglyceride (TG) (mg/dL) median (Q1-Q3)	104 (79.75–135.25)		
Low-density lipoprotein cholesterol (LDL-C) (mg/dL) median (Q1-Q3)	119 (87.25–154.5)		
High-density lipoprotein cholesterol (HDL-C) (mg/dL) median (Q1-Q3)	45 (38–54.75)		
Hypertensives *n* (%)	127 (90.1%)		
Diabetic subjects *n* (%)	45 (31.9%)		
Smokers *n* (%)	62 (44%)		
Obese *n* (%) BMI >= 25	97 (69%)	32 (46%)	
CRP (mg/L) median (Q1-Q3)	2.9 (1.5–6.3)		
Brain lesion size (mm^2^) median (Q1-Q3)	445 (170–923)		
Lesion location (R *n*(%) /L *n*(%) hemisphere)	65 (46.1%)/ 76 (53.9%)		
NIHSS on admission median (Q1-Q3)	8 (6–12)		
NIHSS at discharge median (Q1-Q3)	2 (1–5)		
MRS scale median (Q1-Q3)	2 (1–3)		
HbA1c (%) median (Q1-Q3)	5.9 (5.6–6.5)		
Creatinine (mg/dL) median (Q1-Q3)	0.86 (0.73–1.03)		
Highly sensitive troponin (ng/l) median (Q1-Q3)	5 (5–13.25)		
Fibrinogen (mg/dL) median (Q1-Q3)	375 (325.5–438)		
D-dimer (µg/mL) median (Q1-Q3)	0.86 (0.42–1.425)		
Ejection fraction (EF %) median (Q1-Q3)	56 (52–58)		
Intervention treatment (T ± MT) *n* (%)	60 (42%)		
Trombolysis (T) *n*(%)	48 (34%)		
Mechanical thrombectomy (MT) *n*(%)	24 (17%)		
Hyperlipidemia *n*(%)	102 (72.9%)		
Atrial fibrillation *n*(%)	45 (31.9%)		
Carotid atherosclerosis	115 (81.6%)		
>30% stenosis *n* (%)	30 (21.3%)		
TOAST classification	141 (100%)		
LVD *n*(%)	42 (29.8%)		
SVD *n*(%)	47 (33.3%)		
CE *n*(%)	52 (36.9%)		

Abbreviations: BMI, Body Mass Index. n, Number. M, Male. F, Female. R, Right. L, Left. LVD, Large-vessel disease. SVD, Small-vessel disease. CE, Cardioembolic. Descriptive statistics are presented as number (percentage) for categorical variables and median (1st quartile-3rd quartile) for quantitative variables. * *p*-value of Mann-Whitney test.

**Table 2:** Trace elements status of the study population.

		AIS	Controls	*p* *
Se [μg/L]	Total	57.69 (44.13–70.95)	75.48 (66.33–92.67)	<0.001
	Males	59.71 (42.04–73.14)	75.48 (61.23–99.3)	
	Females	55.65 (44.39–67.98)	75.15 (69.66–87.02)	
	*p* **	0.730	0.931	
Zn [mg/L]	Total	0.62 (0.51–0.73)	0.79 (0.71–0.89)	<0.001
	Males	0.65 (0.51–0.73)	0.76 (0.69–0.88)	
	Females	0.59 (0.51–0.73)	0.79 (0.72–0.90)	
	*p* **	0.573	0.658	
Cu [mg/L]	Total	0.99 (0.82–1.12)	0.97 (0.86–1.24)	0.283
	Males	0.97 (0.8–1.11)	0.86 (0.78–1.11)	
	Females	1.01 (0.84–1.14)	1.06 (0.91–1.29)	
	*p* **	0.245	0.008	
Cu/Zn molar ratio ***	Total	1.68 (1.22–2.09)	1.34 (1.08–1.66)	<0.001
	Males	1.61 (1.22–2.06)	1.19 (1.01–1.41)	
	Females	1.74 (1.20–2.20)	1.46 (1.14–1.76)	
	*p* **	0.485	0.022	
Cu/Se molar ratio ***	Total	21.97 (15.24–29.97)	16.40 (13.73–20.95)	<0.001
	Males	21.45 (15.17–28.28)	13.88 (12.07–20.76)	
	Females	22.33 (15.19–31.25)	17.12 (14.94–21.29)	
	*p* **	0.395	0.037	

Abbreviations: AIS, patients with acute ischemic stroke. Se, Selenium. Cu, Copper. Zn, Zinc. M, Male. F, Female. Descriptive statistics are presented as median (1st quartile-3rd quartile). Normal range Trace Elements [44]: Se (66–104 μg/L), Zn (0.7–1.3 mg/L), Cu (0.7–1.6 mg/L). * comparison of AIS vs Controls, Mann-Whitney test. ** comparison of Males vs Females, Mann-Whitney test. *** population-based reference values have not yet been established.

## Data Availability

The data presented in this study are available on request from the corresponding author.
